# Successful Neuromuscular Blockade Reversal Using Sugammadex and Neostigmine Failed to Prevent Myasthenic Crisis After Emergency Surgery: A Case Report

**DOI:** 10.7759/cureus.27366

**Published:** 2022-07-27

**Authors:** Daniel Haddad, Adeeb J Hanna, Lori Russo

**Affiliations:** 1 Medicine, Rowan University School of Osteopathic Medicine, Stratford, USA; 2 Anesthesiology, North American Partners in Anesthesia (NAPA), Hackensack Meridian Ocean Medical Center, Brick, USA

**Keywords:** post-operative management, myasthenic exacerbation, emergency surgery, sugammadex, neostigmine, neuromuscular blockade (nmb), myasthenia gravis (mg)

## Abstract

Recent literature suggests that the use of sugammadex for the reversal of neuromuscular blocking agents (NMBAs) reduces the risk of postoperative myasthenic crisis (MC) in patients with myasthenia gravis (MG), particularly after thymectomy, but studies are lacking on emergency surgeries. We achieved successful intraoperative reversal of neuromuscular blockade (NMB) using a combination of sugammadex and neostigmine (with glycopyrrolate). However, MC was not avoided and reintubation was required on postoperative day 1.

A 65-year-old male with a longstanding history of MG presented to the emergency department with complaints of abdominal pain, diarrhea, vomiting, chills, and fatigue for three days. A computed tomography (CT) scan of the abdomen showed acute appendicitis, for which he underwent a laparoscopic appendectomy on hospital day 1. The patient received successful general anesthesia with a rapid sequence induction using a smaller than average dose of rocuronium, given his history of MG. At the conclusion of the case, sugammadex followed by neostigmine/glycopyrrolate and a subsequent dose of sugammadex were given, with reversal of NMB. The patient was successfully extubated but required reintubation on postoperative day 1 for hypercapnic respiratory failure.

Our case report on this patient with MG yields two topics that have not been extensively examined. First, dual therapy with sugammadex and neostigmine/glycopyrrolate may provide significant clinical benefit over individual therapy for NMBA reversal, given that their mechanisms of action are different and particularly when sugammadex is given prior to neostigmine/glycopyrrolate. Second, anesthesia literature is lacking on MG patients undergoing emergency surgeries. While sugammadex has been promising in medically optimized non-emergent surgeries, we discuss here a case where sugammadex failed to prevent MC in the emergency surgery setting and a look into what may provide patients with the best chance for avoiding postoperative MC.

## Introduction

Myasthenia gravis (MG) is the most common primary disease affecting the neuromuscular junction (NMJ), with a prevalence of 150 to 250 cases per one million persons. The disease is characterized by autoantibodies that target postsynaptic acetylcholine receptors at the NMJ. This induces progressive generalized or localized muscle weakness with use that classically presents with diplopia, ptosis, and marked proximal muscle weakness.

One of the most feared complications of MG is myasthenic crisis (MC), a severe exacerbation of the disease that manifests as muscular weakness, particularly of those involved in respiration. Consequently, the patient may experience respiratory distress leading to respiratory failure. MC may be triggered postoperatively by infection, emotional stress, medications, and other pathophysiologic changes to the body. Medications that are used frequently in the perioperative period and may incite MC include macrolide and fluoroquinolone antibiotics, class 1a antiarrhythmics, magnesium, and corticosteroids [1]. It has also been shown that neuromuscular blocking agents (NMBAs) such as rocuronium contribute to the development of postoperative MC.

Acetylcholinesterase inhibitors (AChIs) are used in the long-term maintenance of patients with MG. Steroids, intravenous immunoglobulin (IVIG), and plasmapheresis are all modalities used in the treatment of MC. However, overuse of AChIs, particularly with those on high-maintenance doses, may result in a cholinergic crisis that can be difficult to distinguish from MC [2]. The use of AChIs for reversal of neuromuscular blockade (NMB) is a traditional practice for MG patients requiring non-depolarizing agents such as rocuronium. However, the more recent development and increasingly widespread use of sugammadex has shown to be promising in reversing NMBAs. Sugammadex is a modified γ-cyclodextrin that reverses the effects of steroidal NMBAs, with the highest affinity for rocuronium. Its mechanism of action is via binding and inactivating unbound rocuronium in the bloodstream, forming tight complexes, and promoting urinary excretion of the inactivated complex. It has no effect on acetylcholine or acetylcholine levels; thus, there is no risk of cholinergic crisis and limited cholinergic adverse effects such as bradycardia, bowel cramps, and bronchospasm. This makes concomitant administration of an anticholinergic drug such as glycopyrrolate or atropine unnecessary [2]. A multitude of studies have displayed the superior effectiveness of sugammadex over neostigmine for reversal of steroidal NMBAs in MG patients [3-5]. Intraoperatively, sugammadex has a more rapid onset of rocuronium reversal than does neostigmine at any blockade depth [2]. It is also associated with fewer adverse effects, respiratory adverse outcomes, and cardiovascular adverse outcomes compared to neostigmine use. A multicenter study by Kheterpal et al. [6] showed a 30% reduction in pulmonary complications, 47% reduction in pneumonia risk, and 55% reduction in respiratory failure risk compared to neostigmine. Likewise, a retrospective observational study by Mouri et al. [7] found that sugammadex was associated with a reduction in postoperative MC in MG patients undergoing thymectomy with an odds ratio of 0.48% and that total hospitalized costs were also reduced. Sugammadex works only to bind and remove steroidal NMBAs from circulation. AChIs, on the other hand, increase the amount of available acetylcholine at the NMJ to overcome the steroidal non-depolarizing agents that are bound to the abnormal acetylcholine receptors. We suggest that the two classes of reversal agents, γ-cyclodextrins (sugammadex) and AChIs (neostigmine), may provide additive or synergistic positive effects for patients with weakness associated with their MG when given as dual therapy; particularly if receptors are first cleared of NMBA with sugammadex, then acetylcholine levels are increased with neostigmine if reversal is inadequate and weakness persists. Of note, however, careful patient selection would be required, as those on high doses of maintenance AChIs are at risk of cholinergic crisis from the use of neostigmine. More research in the form of structured studies would be beneficial, as would the additional study of emergency surgical cases such as this one.

## Case presentation

**Figure 1:**
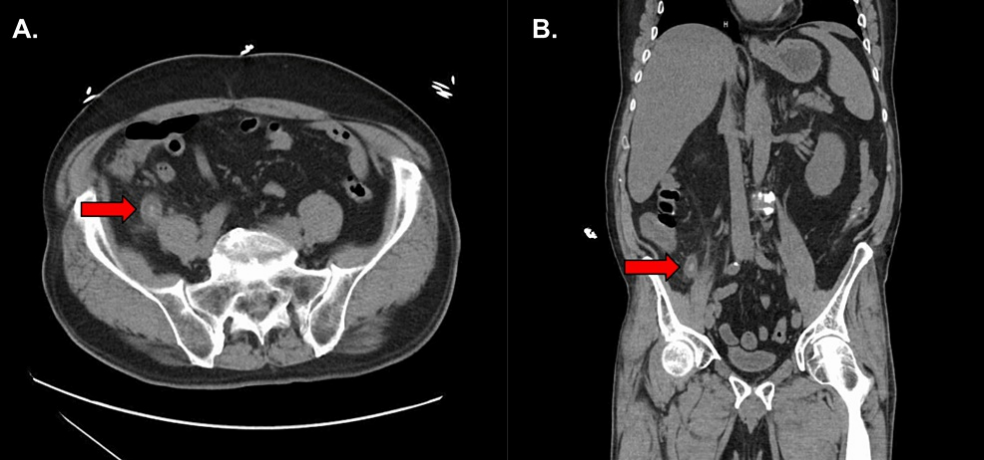
Preoperative CT revealing suspected appendicitis. Axial (A) and coronal (B) CT scan of abdomen and pelvis demonstrating periappendiceal inflammation and the classic target sign of appendicitis (red arrow). CT, computed tomography

A 65-year-old male with a three and a half years’ history of MG presented to the emergency room with three days of abdominal pain, diarrhea, vomiting, chills, and fatigue. His past medical history included paroxysmal atrial fibrillation, type 2 diabetes mellitus, stage II chronic kidney disease, hypertension, and hyperlipidemia, and he was a former smoker. He weighed 91.2 kg and was 182.9 cm tall. The patient’s EKG in the emergency room showed normal sinus rhythm with a prolonged QT. His home maintenance regimen for his MG was pyridostigmine 60 mg QID. He was not being treated with corticosteroids, IVIG, or plasmapheresis, and had no history of thymectomy. His next of kin reported compliance issues with his medications, including those for MG. The patient was brought to the preoperative bay for preparation and evaluation by the anesthesia team on hospital day 1 as an emergent case. On the anesthesiologist’s evaluation, the patient was noted to have garbled speech, which the patient reported was his baseline and secondary to his MG. He did report missing a dose of his pyridostigmine during his acute illness. The patient also reported that he had been vomiting on the day of surgery; therefore, the decision was made to perform a rapid sequence induction. Given the need for paralysis in the setting of a laparoscopic surgical approach and the unpredictability of succinylcholine in MG patients (likely due to their limited availability of ACh receptors), rocuronium was administered at a reduced amount, as MG patients often do not require significant NMBA. A dose of 30 mg was chosen and was successful in achieving intubation quickly without the need for ventilation. The patient was in atrial fibrillation with rapid ventricular response (RVR) prior to induction, with sustained heart rates of approximately 145 beats per minute. He was hydrated and given intravenous diltiazem via bolus (three boluses of 5 mg), which resulted in a conversion back to normal sinus rhythm. A balanced anesthetic of 0.5 MAC of desflurane with a low-dose propofol drip was used to maintain anesthesia. He did require a phenylephrine drip for hypotension, with doses ranging from 40 mcg/min to 100 mcg/min, but was weaned off after rehydration with lactated ringers. For pain control, he received a one-time dose of 100 mcg of fentanyl. The surgeons noted that the patient’s appendix was gangrenous (Figure 1), but there were no significant surgical events during the case.

After the conclusion of the surgery, the train of four (TOF) was 0/4 using a standard twitch monitor. Subsequently, 500 mg (5.48 mg/kg) of sugammadex was administered. Following this, the patient was monitored for 10 minutes but was only achieving tidal volumes of 200 cc (2.19 cc/kg). A dose of 4 mg of neostigmine in combination with 0.4 mg of glycopyrrolate was given. The tidal volumes improved to approximately 500 cc (5.48 cc/kg). While the patient could follow all commands, perform sustained head lift, and had a strong hand grip, he still appeared to be using accessory muscles of respiration. Given that the standard TOF monitor is somewhat subjective and higher doses of sugammadex have been safely used in the literature [8], a second dose of 500 mg of sugammadex was elected to be given approximately five minutes after the neostigmine. The patient continued to show signs of improvement with resolution of accessory respiratory muscle use. He was successfully extubated and placed on bilevel positive airway pressure (BiPAP) shortly after. The intensivists were consulted and assumed care of the patient. Later in the evening, he was showing signs of clinical improvement, and thus the decision was made to downgrade from BiPAP to nasal cannula. On postoperative day (POD) 1, the patient went into atrial fibrillation with RVR, showed signs of acute kidney injury, and became further thrombocytopenic (platelet count went from 101,000 per mcL preoperatively to 30,000 per mcL). This was suggestive of progression to severe sepsis. He was noted to have become lethargic as well, and an arterial blood gas sample yielded a pCO2 of 126.1 mmHg and a pO2 of 89.8 mmHg. He was reintubated due to hypercapnic respiratory failure and started on intravenous steroids and IVIG therapy for MC. The patient was extubated on POD 4 and reintubated for the second time on POD 6, and then extubated for the final time the following day on POD 7. He ultimately improved and was discharged from the hospital on POD 12.

## Discussion

While the etiology of the MC in this patient was multifactorial, sepsis/infection played a significant role in combination with the need for surgery and anesthesia. Exacerbation of MG is commonly caused by stress, infection, and certain medications [9], which were all present perioperatively. The patient was in atrial fibrillation with RVR immediately preceding the operation and was found to have a gangrenous appendix intraoperatively, both of which presumably played a role in the development of MC. Several medications have also been associated with exacerbation of MC. Piperacillin-tazobactam was the antibiotic given and is not known to precipitate MC. However, diltiazem was necessary to return the patient to a physiologically stable heart rate and beta blockers were used within a 24-hour period of surgery as well. Both antidysrhythmics increase the risk of MC [9]. These conditions together potentially contributed to the exacerbation of MG and postoperative development of MC.

The end goal of adequate reversal of NMB is to prevent unwanted downstream effects such as remaining intubated or having prolonged muscle weakness postoperatively. This is especially important in the MG population since they are prone to respiratory failure. The γ-cyclodextrin NMB reversing agent sugammadex binds free NMBA (such as rocuronium) in the circulation, creating a concentration gradient by which rocuronium is released from the receptors. Once the majority of the rocuronium is bound by sugammadex in a patient with MG, the postsynaptic receptors, albeit abnormal ones, become available to the interaction with acetylcholine. Neostigmine, on the other hand, inhibits the enzyme that metabolizes acetylcholine, thereby increasing the amount available at the postsynaptic receptor. A study on NMB reversal with sugammadex by Schaller and Fink [5] reported that the mean time of reversal to a TOF of 0.9 is three minutes. Additionally, a study by de Boer et al. [8] revealed that specifically in the MG patient population, reversal from rocuronium was achieved in four minutes with the relief of residual paralysis and return of TOF to preoperative levels using doses ranging from 0.5 to 12 mg/kg. Sugammadex has been demonstrated to provide rapid and complete reversal of NMBAs in patients of varying degrees of NMB. In MG patients specifically, its use has been associated with lower incidence of postoperative MC as well as shorter and less costly hospital stay post-surgery after elective surgeries [7].

Here we present a case in which the patient was not optimized at baseline for his MG due to compliance issues and arrived for emergency surgery with ongoing acute issues, such as atrial fibrillation with RVR and the onset of sepsis. Given the high likelihood of pulmonary complications if MG patients remain intubated postoperatively, it was imperative that the anesthesia team allow the patient the best chance possible for a successful extubation. After an initial dose of sugammadex, the patient had returned his TOF to 4/4, but clinically the patient was showing evidence of inadequate tidal volumes. This was not conducive to a safe extubation. Clinical improvement was seen after the use of neostigmine, followed by an additional dose of sugammadex as tidal volumes improved. While further structured studies would be needed to test this hypothesis, we believe that dual therapy with sugammadex preceding neostigmine may lead to better outcomes than sugammadex alone or neostigmine alone, particularly in those with underlying muscular weakness. AChIs are widely used for the treatment of MG because they increase the amount of acetylcholine available at the NMJ. It is imperative to optimize the interactions of acetylcholine with the remaining acetylcholine receptors that are not inhibited by autoantibodies. Patients with MG undergoing procedures requiring NMBAs may benefit from adequate reversal with sugammadex followed shortly by neostigmine. The reversal of NMBA with sugammadex combined with MC reversal with neostigmine may provide a synergistic mechanism that improves postoperative outcomes in patients with MG. Additionally, large doses of sugammadex have not been shown to be harmful [8]. Larger doses may be useful in patients with MG or other conditions resulting in skeletal muscle weakness, as we again saw clinical improvement in the form of reduced use of muscles of respiration following a second dose.

MG patients have unpredictable responses to sugammadex reversal in some studies. One case review showed 13 successful NMB reversals with sugammadex, but four failed ones. Notably, of the four failures, one reversal proved successful following subsequent neostigmine delivery [10]. Similarly, an additional case report showed successful NMB reversal with neostigmine after sugammadex failed. A randomized controlled trial showed similar, improved outcomes in groups receiving either 2 mg/kg of sugammadex or 1 mg/kg of sugammadex plus 50 µg/kg of neostigmine as opposed to 1 mg/kg of sugammadex given by itself [11]. Additionally, a case report demonstrated that a half dose of sugammadex combined with neostigmine was noninferior to a full dose of sugammadex [12]. These studies demonstrate a potential benefit of dual therapy that may be equal or superior to either class alone.

The improved comfort after the second dose of sugammadex, despite having TOF of 4/4, may be in part due to the fact that the TOF monitors are subjective and it is feasible that there was residual NMB. TOF monitoring was heavily relied upon in the care of this patient. Peripheral nerve stimulation and monitoring via TOF is useful for monitoring depth of NMB as well as recovery and appropriate timing of medication. However, TOF does have limitations. It is unable to detect fade when TOF ratios are above 0.4, meaning that continued blockade may be present despite TOF findings in ratios between 0.4 and 0.9. This can lead to erroneous premature extubation of a patient [13]. Twitch response at the orbicularis oculi muscle, although potentially overestimating persistent NMB, may be useful for avoiding resistant NMB in patients with MG [14]. A rat model study demonstrated in Takahashi et al.’s paper found that the severity of the MG limited the reliability of the TOF monitoring used in recovery evaluation. In the study, rats were divided into groups to differentiate the severity of the disease based on clinical findings. TOF monitoring performed in these different groups compared TOF ratio and T1 of TOF. It was found that while T1 of the TOF decreased to the same extent in severe and moderate groups, the TOF ratio in the severe group did not decrease to the same extent that the moderate group value did. The researchers concluded that the discrepancy in TOF ratio changes can cause an overestimation of recovery from NMB [15]. In our patient, despite unchanged TOF ratios, an obvious clinical improvement was noted following 4 mg of neostigmine and the second dose of sugammadex. This unreliability of the TOF monitoring could possibly be attributed to the patient's known MG diagnosis or the limitations of TOF monitoring as a general tool. Therefore, use of a quantitative TOF monitor may be preferred over subjective no-fade assessment in MG patients.

The case described in this study was seen in an emergency surgical setting with atrial fibrillation and acute findings consistent with MG, in addition to chronic medical conditions. Literature on emergent surgeries in MG patients is scarce and limited to case reports, such as the two cases described by Casarotti et al. [16]. In both studies, MG patients underwent rapid sequence rocuronium induction and subsequent reversal with sugammadex (one for emergent laparotomy and the other for emergent endoscopy for hematemesis). In both cases, rapid reversal was observed with adequate TOF restoration. Both patients were observed for a period of 30-40 minutes to ensure that no residual curarization was present before extubation was performed [15]. Literature supports the use of sugammadex in MG patients receiving non-depolarizing steroidal NMBAs for surgery to prevent postoperative MC. While sugammadex substantially helps the elective surgery population, the mainstay of preventing MC in the emergency surgery population should strongly focus on the enhancement of the patient’s MG regimen long before an emergency occurs and optimizing the patient’s pathophysiology related to their emergency surgery very closely. Improving volume status, preventing or treating infection, controlling physiologic parameters such as heart rate and normal rhythm, and avoiding medications that exacerbate MG are all measures that should be promptly addressed in emerging cases.

## Conclusions

MG can be a debilitating disease process that profoundly affects the strength of muscles throughout the body, particularly those involved in respiration. These patients pose a significant challenge for anesthesia teams requiring NMBAs intraoperatively. In this case report, we showed both clinical and measurable improvement in breathing/ventilation after a repeat dose of sugammadex combined with neostigmine, following an unsuccessful NMB reversal with a single dose of sugammadex. We were able to meet acceptable extubation parameters using this dual therapy, but were unable to prevent a postoperative MC in a patient presenting for emergency surgery. Structured studies are needed to delineate whether anesthesiologists should have a lower threshold to add neostigmine after unsuccessful NMB reversal with sugammadex in patients with MG. Most studies currently available examine MG patients' surgical recovery from elective and planned procedures, in which patients have typically been optimized in their outpatient MG regimen. The majority of the studies in patients with MG receiving sugammadex are lacking in emergent surgical cases such as this one.
